# Comparing Knowledge, Accessibility, and Use of Evidence-Based Chronic Disease Prevention Processes Across Four Countries

**DOI:** 10.3389/fpubh.2018.00214

**Published:** 2018-08-02

**Authors:** Anna J. DeRuyter, Xiangji Ying, Elizabeth L. Budd, Karishma Furtado, Rodrigo Reis, Zhaoxin Wang, Pauline Sung-Chan, Rebecca Armstrong, Tahna Pettman, Leonardo Becker, Tabitha Mui, Jianwei Shi, Tahnee Saunders, Ross C. Brownson

**Affiliations:** ^1^Prevention Research Center in St. Louis, Brown School of Social Work and Public Health, Washington University in St. Louis, St. Louis, MO, United States; ^2^Counseling Psychology and Human Services, Prevention Science Institute, College of Education, University of Oregon, Eugene, OR, United States; ^3^School of Medicine, Tongji University, Shanghai, China; ^4^Department of Applied Social Sciences, The Hong Kong Polytechnic University, Hung Hom Kowloon, Hong Kong; ^5^Melbourne School of Population and Global Health, The University of Melbourne, Melbourne, VIC, Australia; ^6^Department of Physical Education, Federal University of Parana, Curitiba, Brazil; ^7^Washington University School of Medicine, Washington University in St. Louis, St. Louis, MO, United States

**Keywords:** evidence-based practice, prevention, chronic disease, knowledge, public health

## Abstract

**Background:** Evidence-based chronic disease prevention (EBCDP) effectively reduces incidence rates of many chronic diseases, but contextual factors influence the implementation of EBCDP worldwide. This study aims to examine the following contextual factors across four countries: knowledge, access, and use of chronic disease prevention processes.

**Methods:** In this cross-sectional study, public health practitioners (*N* = 400) from Australia (*n* = 121), Brazil (*n* = 76), China (*n* = 102), and the United States (*n* = 101) completed a 26-question survey on EBCDP. One-way ANOVA and Pearson's Chi-Square tests were used to assess differences in contextual factors of interest by country.

**Results:** Practitioners in China reported less knowledge of EBCDP processes (*p* < 0.001) and less use of repositories of evidence-based interventions, than those from other countries (*p* < 0.001). Academic journals were the most frequently used method for accessing information about evidence-based interventions across countries. When selecting interventions, Brazilian and Chinese practitioners were more likely to consider implementation ease while the Australian and United States practitioners were more likely to consider effectiveness (*p* < 0.001).

**Conclusions:** These findings can help inform and improve within and across country strategies for implementing EBCDP interventions.

## Introduction

Chronic diseases are an increasing threat to the health and well-being of individuals and communities in middle- and high-income countries (1), and pose many challenges, including reduced quality of life and productivity (2). Further, the majority of premature deaths worldwide are due to chronic disease, and nearly 80% of chronic disease-related mortality occurs in middle-income countries (3, 4).

Similar to evidence-based medicine, evidence-based public health (EBPH), more specifically, evidence-based chronic disease prevention (EBCDP) has proven to be an effective set of processes for reducing the burden of chronic disease. These processes may vary, but include ensuring that decision-makers have up-to-date scientific evidence about the chronic disease prevention policies and programs that are effective at improving chronic disease health outcomes, using that evidence to inform health and policy agendas, and considering the environments and contextual circumstances likely to impact the application of chronic disease prevention evidence (5–7). When EBCDP is applied, it can prevent many cases of morbidity and mortality due to chronic disease (8). However, the implementation of evidence-based interventions can vary across countries due to economic, political, structural, and sociological contextual factors (9). Studies conducted in high-income countries (e.g., Australia, Canada, and the United States) have shown that knowledge of EBCDP processes, and access to evidence-based interventions, positively influence the implementation of EBCDP (10). However, there is a lack of evidence-base regarding these fundamental factors (i.e., knowledge, access, use) in middle-income countries, and how these factors compare with EBCDP in high-income countries. More research is needed to better understand how knowledge of EBCDP processes and access to evidence-based interventions may vary across middle- and high-income countries, to inform and improve future strategies for implementing EBCDP interventions and reducing chronic diseases worldwide (11–14).

The objective of this study is to begin to describe similarities and differences in knowledge of EBCDP processes, avenues for accessing information about evidence-based interventions, factors that influence decision-making regarding planning and implementing evidence-based programs, and use of evidence-based repositories and quality improvement processes across Australia, Brazil, China, and the United States. For this study, EBCDP repositories are considered collections of evidence-based, chronic disease-related interventions (e.g., Guide to Community Preventive Services in the United States, Health-Evidence.org in Canada, Cochrane Collaboration in all countries). Likewise, quality improvement processes are defined as ongoing, formal assessments of the effectiveness and quality of public health chronic disease prevention efforts (15).

For several reasons, Australia, Brazil, China, and the United States were chosen as the countries of interest for this study. First, they have a high prevalence of chronic diseases (e.g., these four countries are estimated to contribute to 32.9% of the total global burden of cancer) (16, 17). Additionally, these four countries have notably different levels of peer-reviewed EBCDP empirical literature; China and Brazil have far less EBCDP literature than Australia and the United States [(13, 18–22)). There is also wide variation in relevant contextual factors across the four countries (e.g., political and economic structures), and they hold positions as thought leaders in their respective regions of the world (23–26).

## Materials and methods

In this cross-sectional study, public health practitioners (*N* = 400) from Australia (*n* = 121), Brazil (*n* = 76), China (*n* = 102), and the United States (*n* = 101) completed a 26-question survey on EBCDP implementation. Seven questions from this survey were used in this analysis (see Appendix). This survey was conducted to assess contextual factors that influence the implementation of EBCDP in Australia, Brazil, China, and the United States. The 26-question survey was informed by the following: the development of a guiding framework based on previous work of the research team (27, 28); a literature review of EBCDP measures to identify relevant questions and gaps (2, 27, 29–33); and semi-structured interviews of public health practitioners working in chronic disease prevention in Australia (*n* = 13), Brazil (*n* = 9), China (*n* = 16), and the United States (*n* = 12). Drafts of the survey were reviewed by 13 chronic disease prevention researchers, and were translated forward and backward to Chinese and Portuguese from English. The survey was also pilot-tested in each country to ensure contextual appropriateness. Seven response items were deemed non-applicable for China contexts, but were included in the survey for the other three countries.

The team members in each country recruited public health practitioners working in chronic disease prevention, primarily on the local and regional levels, in each of the four countries, to complete the survey. Convenience samples of practitioners were identified through national databases and networks of chronic disease prevention practitioners between November 2015 and April 2016, and recruitment took place via phone and email. For public databases, permission was not requested, but for networks and databases where permission was required, it was obtained. All surveys were delivered by an email embedded link and completed electronically. Upon completion of the survey, all respondents were asked to re-take the survey 2–3 weeks later for test-retest reliability testing purposes, which was repeated until each survey respondent had been contacted twice, with a request to retake the survey. The survey assessed five stages of dissemination (i.e., innovation, awareness, adoption, implementation, and maintenance), five levels of contextual factors (i.e., individual, organizational, community, sociocultural, and political/economic factors), and demographic information of the practitioners (27, 28). Public health systems across the four sampling frames varied so greatly that there was no directly equivalent sampling method that fit the context of all four countries, and thus response rates varied across countries. Australia's response rate was 18%, Brazil's was 46%, China's was 87%, and the United States' was 58%. All practitioners provided informed consent before beginning the survey. This study was approved by the Washington University Institutional Review Board, and the research ethics committees at The University of Melbourne, Pontifical Catholic University of Parana, and Hong Kong Polytechnic University.

### Survey questions

Level of knowledge on evidence-based processes was assessed with the following definition and question, “Evidence-based public health is defined as: The process of integrating science-based interventions with community preferences to improve the health of populations. With this definition in mind, how knowledgeable are you with evidence-based processes?” with response options including, not at all knowledgeable, slightly knowledgeable, somewhat knowledgeable, moderately knowledgeable, and extremely knowledgeable.

The channels that participants used for accessing evidence-based interventions were assessed by asking “Which avenues do you use to learn about the current study findings on evidence-based interventions?” The following question was asked to assess which avenues practitioners would like more access to, “For which avenues would you like additional access?” Both questions had the same list of response options. Respondents were prompted to select all response options that apply.

Factors that influence decision-making were measured by the question, “When you make decisions about such things as program planning and implementation, which of the following are important to you?” Respondents were given a list of 12 possible factors, and prompted to select the top three that were most influential in their decisions to select an intervention.

Use of repositories by self and other staff at respondents' workplace was assessed by defining repositories and asking practitioners to complete the two versions of this statement, “I/Staff have used repositories to find evidence-based interventions…” The following response options included, in none of my/their programmatic areas, in a few of my/their programmatic area, in many of my/their programmatic areas, in all of my/their programmatic areas, I don't know, and not applicable.

Use of quality improvement processes by staff at the respondents' workplaces was assessed by defining quality improvement processes and asking practitioners to complete the following statement, “Staff at my agency use quality improvement processes…” Response options included, in none of my/their programmatic areas, in a few of my/their programmatic areas, in many of my/their programmatic areas, in all of my/their programmatic areas, I don't know, and not applicable. In **Table 2**, we present a truncated version of the survey results, where we calculated a weighted average for questions that were scalable, and presented the top response for categorical, non-scalable questions. The full list of questions included in this analysis, and subsequent results, can be found in Appendix. See Table 3 for the full list of the 26-question survey, including those not included in the analysis for this study.

### Analyses

All analyses were performed using SPSS version 23 (34). Descriptive statistics were performed to determine participant demographic characteristics. One-way ANOVA was conducted for the question with a 5-point Likert scale (EBCDP Knowledge), and Pearson's Chi-Square test was employed for questions with four or fewer response options (Access, Decision-Making, Use of Repositories, and Use of Quality Improvement Processes). The Shapiro-Wilk test was performed to determine normality of the five-level variables, and the Kruskal-Wallis test was used as an alternative to ANOVA if the assumption of normality was violated (35).

## Results

Across the four countries, 400 public health practitioners working in chronic disease prevention completed the survey (Table 1). Most of the practitioners from Australia, Brazil, and the United States worked in local health departments, whereas the majority of practitioners from China were physicians, and worked in community hospitals. Across all countries, the majority of practitioners were female (66–88%). Practitioners were younger in Australia and China than in Brazil and the United States. In Australia, Brazil, and the United States, most practitioners had a Master's degree or higher (36–57%). Australia had the largest percentage of practitioners (42%) working at organizations with more than 400 employees, while the United States had the largest percentage (57%) of practitioners working at organizations with 100 or fewer employees.

**Table 1:** Descriptive statistics of the study sample of chronic disease prevention practitioners in Australia, Brazil, China, and the United States, 2015–2016, *N* = 400.

**Variable**	**Australian(%)**	**Brazil n(%)**	**China n(%)**	**United States n(%)**
Total n	121	76	102	101
Female	107(88)	50(66)	71(70)	88(88)
**AGE**				
21–29	25(21)	6(8)	22(22)	7(7)
30–39	40(33)	28(37)	58(57)	22(22)
40–49	18(15)	23(30)	11(12)	29(30)
50–59	25(21)	16(21)	4(4)	29(30)
≥60	13(11)	3(4)	0(0)	11(11)
**DEGREE**				
Doctorate	17(14)	3(4)	0(0)	7(7)
Master's	51(43)	24(32)	24(24)	49(49)
Bachelor's	36(30)	17(23)	70(69)	28(28)
Other	16(13)	31(41)	8(7)	16(16)
**NUMBER OF WORKPLACE EMPLOYEES**				
0–100	43(38)	27(38)	10(10)	57(57)
101–400	23(20)	20(28)	66(67)	25(25)
>400	48(42)	24(34)	22(22)	18(18)
**SIZE OF JURISDICTION SERVED**				
0–49,999	30(28)	20(29)	5(6)	24(24)
50,000–99,999	11(10)	7(10)	10(12)	25(25)
100,000–399,999	22(21)	19(28)	50(60)	26(26)
>400,000	43(41)	22(32)	19(23)	24(24)

### Knowledge of evidence-based processes

Differences in knowledge of evidence-based processes, and use of and access related to evidence-based interventions, are outlined in Table 2 and Appendix. Mean self-assessed knowledge of evidence-based processes differed significantly across countries. On average, practitioners from China reported less knowledge compared with practitioners from Australia, Brazil, and the United States (Mean = 2.59, 3.84, 3.71, and 4.05 respectively, *p* < 0.001).

**Table 2:** Knowledge, use, and access related to evidence-based interventions in Australia, Brazil, China, and the United States in 2015–2016 (short version).

**Variable**	**Australia (*n* = 121)**	**Brazil (*n* = 76)**	**China (*n* = 102)**	**United States (*n* = 101)**	***P*-value**
Knowledge of evidence-based chronic disease prevention processes (M ± SD)	3.84 ± 0.8	3.71 ± 0.9	2.59 ± 1.0	4.05 ± 0.8	<0.001
**TOP FACTOR INFLUENCING DECISION-MAKING ABOUT PROGRAM PLANNING AND IMPLEMENTATION**					
Support from leadership at my agency			64(63%)		<0.001
Available resources (program dollars and staff)		69(91%)		63(62%)	<0.001
Evidence regarding the effectiveness of the intervention	89(74%)				<0.001
Weighted average extent of use of repositories to find evidence-based interventions	64%	77%	47%	65%	<0.001
Weighted average extent of other workplace staff who use repositories to find evidence-based interventions	25%	66%	42%	54%	<0.001
Weighted average extent of other workplace staff who use quality improvement processes	69%	63%	49%	64%	<0.001
**TOP AVENUE USED TO ACCESS EVIDENCE-BASED INTERVENTIONS**					
Academic journals	107(88%)		57(56%)		<0.001
Conferences				70(70%)	<0.001
Internet search engines		43(57%)			<0.001
**TOP AVENUE FOR WHICH ADDITIONAL ACCESS IS NEEDED**					
Academic journals	42(40%)				0.06
Conferences		42(55%)			<0.001
Evidence-based repositories			38(37%)	32(35%)	0.12

**Table 3:** Factors influencing the dissemination and implementation of evidence-based chronic disease prevention across four countries: an assessment tool.

**Questions**	**Response options**
**AWARENESS**	
Evidence-based public health is defined as: “the process of integrating science-based interventions with community preferences to improve the health of populations” (8). With this definition in mind, how knowledgeable are you with evidence-based processes? (*select one*)	Not at all knowledgeable Slightly knowledgeable Somewhat knowledgeable Moderately knowledgeable Extremely knowledgeable
**ADOPTION**	
Definition: Evidence-based interventions are those that several studies have found to be effective at preventing chronic disease. Repositories are collections of evidence-based interventions [e.g., Guide to Community Preventive Services) (US), Health-Evidence.org (Australia), Cochrane Collaboration (US, Australia)]. 2. I have used repositories to find evidence-based interventions: (*select one*)	in none of my programmatic areas in a few of my programmatic areas in many of my programmatic areas in all of my programmatic areas
3. Staff at my agency use repositories of evidence-based interventions: (*select one*)	in none of my programmatic areas in a few of my programmatic areas in many of my programmatic areas in all of my programmatic areas
4. When you make decisions about such things as program planning and implementation, policy development, or funding, which of the following are important to you? (*select the top three*)	Support from leadership at my agency Support from elected officials Support from community partnerships Recommendations from the funding agency Colleagues are using the intervention Available resources (program dollars and staff) How easy the intervention or policy is to implement Evidence regarding the effectiveness of the intervention Health planning tools (e.g., MAPP or Health People 2010) Relevance of the intervention to the population of interest Seriousness of the health problem Other, please specify ______ Not applicable
5. What avenues do you use to learn about the current study findings on evidence-based chronic disease prevention interventions? (*select all that apply*)	Academic journals Conferences Email alerts Evidence-based repositories Facebook Funders ^a^ Government agency staff Government reports Internet search engines Listservs/Newsletters/Online forums Media campaigns/Media interviews Networks Partnerships (e.g., with universities, health departments, professional associations) Policy briefs^a^ Press releases Stakeholders^a^ Technical assistance/Data liaison Trainings/Workshops/Meetings within my agency Webinars Other, please specify ______ None
6. For which avenues would you like additional access? (*select all that apply*)	*Same responses as #13*
**IMPLEMENTATION**	
7. Approximately what percentage of programs supported by your agency would you say are evidence-based?	*Fill in the blank 0–100%*
8. As you think about the future, what is one thing you would change to help you implement evidence-based chronic disease prevention interventions?	*Fill in the blank*
**MAINTENANCE**	
Quality improvement (QI) refers to ongoing formal assessments of the effectiveness and quality of public health chronic disease prevention efforts. 15. Some examples of quality improvement processes include: Results-based accountability (RBA), Community Health Improvement Plan (CHIP), Plan-Do-Study-Act (PDSA), and Plan-Do-Check-Act. 9. Staff at my agency use quality improvement processes: (*select one*)	in none of my programmatic areas in a few of my programmatic areas in many of my programmatic areas in all of my programmatic areas
10. In your opinion, how often do programs end that should have continued? (i.e., end without warrant) (*select one*)	Never Sometimes Often
11. When you think about public health programs that have ended, what are the most common reasons for programs ending? (*Select the top three*)	Program was never evaluated Program was evaluated but did not demonstrate impact Opposition/lack of support from leaders in my agency Opposition/lack of support from the general public Opposition/lack of support from policy makers Funding diverted to a higher priority program Grant funding ended Change in political leadership Insurance funding/coverage ended Program was adopted or continued by other organizations A program champion departed Program was not evidence-based Program was expensive Program was challenging to maintain Other, please specify ______ I do not know Not applicable
12. In your opinion, how often do programs continue that should have ended? (i.e., continue without warrant) (*select one*)	Never Sometimes Often
13. When you think about public health programs that continued that should have ended, what are the most common reasons for their continuation? (i.e., continue without warrant) (*Select the top three*)	Program was never evaluated Sustained support from leaders in your agency Sustained support from the general public Sustained support from policymakers Prohibitive costs of starting something new Absence of alternative options Sustained funding Presence of a program champion Program was considered evidence-based Program was low-cost Program was easy to maintain Other, please specify ______ I do not know Not applicable
**CONTEXTUAL FACTORS**	
14. Which of the following are personal barriers that make it harder for you to select and implement evidence-based chronic disease prevention interventions? *(Select all that apply*)	Not being an expert on relevant issues Lack of confidence in finding data and statistics Lack of skills to develop evidence-based interventions Lack of confidence in carrying out evidence-based interventions Lack of decision-making authority Low value of evidence-based approaches Workload is too heavy/not enough time Overwhelmed by task Other, please specify ______ None
15. Which of the following are agency-level barriers that make it harder for you to select and implement evidence-based chronic disease prevention interventions? (*Select all that apply*)	Poor understanding of evidence-based approaches Culture/climate is not supportive of change/new ideas No existing policies to support evidence-based approaches Agency does not provide training in evidence-based approaches Staff/leaders lack formal training in evidence-based approaches Lack of access to resources (e.g., computer, Internet) Not enough funding Low priority placed on chronic disease prevention No systems to ensure interventions are evidence-based Not enough staff Beliefs that evidence-based interventions are too difficult to implement/sustain Other, please specify ______ None
16. Which of the following are community-level barriers that make it harder for you to select and implement evidence-based chronic disease prevention interventions? (*Select all that apply*)	Lack of access to repositories/databases of scientific studies Lack of partnership between agency and community Community members' needs compete with evidence-based recommendations Catering to preferences of funders ^a^ Low priority placed on chronic disease prevention Other, please specify ______ None
17. Which of the following are sociocultural barriers that make it harder for you to select and implement evidence-based chronic disease prevention interventions? (*Select all that apply*)	Distrust of scientific data in the populations served Community cultural practices conflict with evidence-based recommendations Not enough relevant evidence for populations served Serving a rural setting where data are lacking ^a^ Serving a highly disadvantaged population Serving a population that speaks a language different from the majority ^a^ Evidence is presented in a language I do not understand Other, please specify ______ None
18. Which of the following are political/economic barriers that make it harder for you to select and implement evidence-based chronic disease prevention interventions? (*Select all that apply*)	Political leaders not providing enough support Funding changes that occur with changes in political leadership Political climate conflicts with evidence-based chronic disease prevention recommendations Health care system does not support evidence-based chronic disease prevention Other, please specify ______ None
19. For which of the following skills would you like additional technical support or training? (*Check all that apply*)	Prioritizing program and policy options Quantifying the public health issue using descriptive epidemiology (e.g., concepts of person, place, time) Using quantitative evaluation approaches (e.g., surveillance or surveys) Using qualitative evaluation approaches (e.g., focus groups, key informant interviews) Developing an action plan for achieving goals Defining the health issue according to the community's needs and assets Adapting interventions for different communities and settings Using economic data in the decision making process Communicating research to policy makers Other, please specify ________ None
**INDIVIDUAL AND AGENCY CHARACTERISTICS**	
20. What is your gender? (*select one*)	Male Female Other Prefer not to answer
21. What is your age? (*select one*)	21–29 30–39 40–49 50–59 60 and over Prefer not to answer
22. What degree/credentials do you hold? (*Check all that apply*)	BS/BA CHES Certified Health Educator (in Diabetes, Asthma, etc.) RN or RD MS or MSc MPH or MSPH MA Other Master's degree NP MO or DO Ph.D., Dr.PH, ScD Other, please specify ______
23. Though you may work in several capacities, how do you best describe your primary position? (*select one*)	Academic Researcher Academic Educator Community Health Nurse Department Head Division or Bureau Head/ Division Deputy Director Epidemiologist Health Educator Nutritionist/ Dietician Physician Program Manager/Administrator/Coordinator Program Planner/ Evaluator Public Health Specialist Social Worker Statistician Other, please specify ______
24. The agency in which I work has the following number of employees. (*select one*)	0–50 51–100 101–200 201–400 401–800 >800 I do not know
25. The size of the population my agency serves is has the following number of people. (*select one*)	0–24,999 25,000–49,999 50,000–74,999 75,000–99,999 100,000–149,999 150,000–199,999 200,000–299,999 300,000–399,999 400,000+ I do not know
26. Is there anything else you would like to share on the topic of evidence-based chronic disease prevention? Please specify.	Fill in the blank

a *This item was not applicable and not included in the survey for respondents in China*.

### Access to evidence-based interventions

Academic journals, conferences, evidence-based repositories, the Internet, partnerships, and trainings were among the most frequently used avenues for accessing evidence-based interventions across all countries. Practitioners from China reported significantly less frequent use of most of the avenues including the Internet, government reports, and government staff relative to all other countries (*p* < 0.001). Compared with both Brazil and China, practitioners in Australia and the United States were significantly more likely to use email alerts, networks, and technology-based avenues including webinars and listservs/email newsletters/online forums (each significant at *p* < 0.001).

When practitioners were asked about avenues to which they needed additional access, the most frequently reported avenues across all four countries included academic journals, conferences, email alerts, evidence-based repositories, partnerships, and training/workshops/meetings within the agency. However, for avenues that practitioners needed additional access to, approximately half were not significantly different across the four countries. Practitioners in Australia requested increased access to professional networks (*p* = 0.001) more frequently than those from the other countries. Similarly, Brazilian practitioners highlighted their desire for additional access through conferences (*p* < 0.001), and practitioners in China cited a need for additional access to Internet search engines (*p* = 0.001) and Facebook (*p* < 0.001) more frequently compared with practitioners from the other countries.

### Evidence-based decision-making

When asked to consider factors that influence their decision-making when planning and implementing evidence-based programs, practitioners from Brazil and China were more likely to report making decisions based on leadership (*p* < 0.001), elected officials (*p* < 0.001), and ease of intervention implementation (*p* < 0.001) compared with American and Australian counterparts. Practitioners from China were significantly less likely to make decisions based on the evidence regarding effectiveness of the interventions (*p* < 0.001), whereas for practitioners from Australia and the United States, evidence was commonly considered (74 and 61% respectively). In Brazil, practitioners were more likely than those from the other three countries to make decisions based on support from community partnerships (*p* = 0.001), recommendations from funding agencies (*p* < 0.001), whether their colleagues are using the intervention (*p* < 0.001), available resources (*p* < 0.001), health planning tools (*p* < 0.001), the relevance of the intervention to the population of interest (*p* < 0.001), and the seriousness of the health problem (*p* < 0.001).

### Use of evidence-based repositories and quality improvement processes

There was a significant difference in the use of evidence-based repositories and quality improvement processes among practitioners across countries. Specifically, the majority of practitioners in China reported repository use and use of quality improvement processes in few or no programmatic areas, whereas the majority of practitioners in all three other countries reported repository use in many or all programmatic areas (*p* < 0.001).

## Discussion

The primary pattern that emerged from the results overall, was the difference in EBCDP between the two high- income (Australia and the United States) and the two middle-income (Brazil and China) countries. Practitioners from both high-income countries tended to have more knowledge, access, and experience, with regard to the implementation of EBCDP, compared with practitioners from both middle-income countries.

Practitioners from Brazil and China reported less knowledge of evidence-based processes than practitioners from Australia and the United States, with practitioners from China reporting the least EBCDP knowledge. This finding is consistent with the literature, citing deficiencies with regard to evidence-based practice in the field of public health in middle-income regions of the world, specifically China (36). In China, the lack of knowledge related to evidence-based processes in chronic disease prevention may be due to the recent, rapid increase in chronic disease compared to infectious disease, while still facing high rates of infectious disease, making it potentially more difficult to learn and adopt the processes of EBCDP (37–41).

Also, postgraduate degrees were less common among practitioners from China and Brazil than among practitioners from Australia and the United States. For practitioners from Brazil, one of the most common degrees was a certificate in public health (less than a bachelor's degree); whereas consistent with the structure of the healthcare system in China, practitioners in this sample tended to have a bachelor's degree in medicine and work as physicians in community hospitals. This reflects the contextual differences between high- and middle-income countries, of practitioners working in the field of chronic disease prevention. Additionally, differences in knowledge between both of the two middle- and high-income countries could be attributed to the former having larger and better funded institutions that have the resources to fund, generate, and disseminate research on EBCDP. Fewer resources can put middle-income countries at a disadvantage when it comes to supporting the integration and adoption of evidence-based practices into the field of public health (42).

Practitioners from China most commonly reported evidence-based repository use by themselves and by colleagues in few to no programmatic areas. One possible explanation is the less established nature of EBCDP in China, supported by a study conducted in China where researchers examined EBCDP in western countries, to inform EBCDP in China, finding that the developed western countries had a much larger evidence base for chronic disease prevention, compared to China, whose chronic disease prevention evidence base was behind due to less theoretical guidance and intervention measure development (43). Further, practitioners with low levels of knowledge relating to evidence-based processes may not be aware of how to find relevant repositories of evidence-based information, or how to use them to attain the information they need. Moreover, in the United States and Australia, where repositories were reportedly used more often, other contextual factors may underlie these patterns. For example, Australia practitioners report valuing networks as a way of sharing and learning about new evidence-based initiatives (44). This information-sharing may contribute to both increased knowledge of evidence-based processes, and repositories and/or knowledge of their colleagues' use of repositories. Further, high-income countries were more likely to employ avenues that required previous knowledge of EBCDP and where to find information about it, such as email alerts and online resources. Using networks as a primary source for identifying evidence-based interventions implies that the individuals in Australia that make up that network are valuable resources for such information.

The two middle-income countries were more likely to value ease of intervention implementation when making decisions, which similarly could be a result of a less established commitment to EBCDP and fewer resources dedicated to this area. Similarly, China was less likely to consider the evidence regarding effectiveness of interventions when making decisions, which also aligns with their higher value on ease of implementation. This contrasts with the two high-income countries who placed high value on effectiveness of intervention and low value on ease of intervention. One potential explanation is that evidence bases produced in other countries, such as the United States, might not be applicable or transferable to countries like China, making the research base, regardless of its size, of low value to Chinese practitioners. Further, this finding may reflect the systems and policies set up for supporting and/or requiring evidence-based interventions in high-income countries (e.g., funders and administrators requiring the use of evidence-based interventions, access to evidence, etc.). For example, in a United States study mapping the landscape of behavioral health interventions, it was found that funders of such interventions required 75% of all money spent by a program funded under the initiative be spent on evidence-based programming (45). If using evidence-based interventions is not required, then choosing ease of intervention would make logical sense in middle-income countries.

Similarities across all countries also arose. Both of the two high- and middle-income countries most often used academic journals and conferences as avenues for accessing new evidence, which is consistent with literature on best practices in evidence-based public health (EBPH), directing readers to academic journals as the primary avenue for finding relevant evidence (18). However, as is shown in the literature on barriers to evidence-based practice, among practitioners who do not have access to academic journals, the internet is a common, accessible source for finding evidence (46, 47), which is reflected in this study's findings; both high- and middle income countries frequently cited the internet as a common avenue for accessing evidence.

## Limitations

This study has several limitations to consider. Because the types of public health practitioners are not equivalent across the countries in this study, it is hard to control for this variable. Additionally, non-random sampling, as carried out in this study, is susceptible to selection bias, and there may be distinct differences between those who were selected and opted to take this study's survey compared with those who were not selected or opted against taking the survey. This study also did not control for potential confounders like age, job responsibilities, and culture. While the study did ask about many of these variables, small cell sizes precluded multivariable analyses in this study. Future studies should strive to have a large enough sample size to include all potentially confounding variables in the analysis. Further, because some of the chi-square analyses had cells where expected values were < 5, these results should be cautiously interpreted, even when statistically significant, and a larger, more representative study of public health practitioners in low-, middle-, and high- income country is needed, to validate study findings. Additionally, future studies should conduct country-specific research on similar variables, to validate findings from this study, and deepen the evidence-base in this area. Doing so will enable researchers to better understand, explain, and address the contextual similarities and differences of practitioner knowledge, access, and use of EBCDP, among other key contextual variables.

Additionally, while this study focuses on the value of EBCDP in reducing chronic disease, the current evidence-base also supports several limitations of evidence-based practice across many fields (48–50), which should be acknowledged in this study. These limitations include a lack of scientific evidence available to practitioners to inform their practice; research available not being relevant to the populations that practitioners are serving; the definition of EBCDP being too narrow and restricting practices that might prove to be effective for practitioners and their clients; and the cost of conducting exclusively rigorous studies to develop an evidence base could be cost prohibitive, especially for middle- and low-income countries. Therefore, future studies examining contextual factors that influence EBCDP knowledge, access, and use across multiple countries should consider incorporating a broader terminology, such as evidence-informed decision-making (EIDM), to recognize the value of multiple types of evidence. Evidence-informed decision-making is defined as the use of research evidence, practitioner expertise, existing public health resources, and knowledge about the local community and political climate, and promotes practitioners using multiple types of information, not just scientific evidence, to make comprehensive, informed decisions to address their clients' needs (44, 51–53). Further, it is possible that using EBCDP as the lens for this study, could explain some of the variances between practitioners from middle- and high-income countries. Future studies should consider examining cross-country differences and similarities across EIDM knowledge, access, and use.

## Conclusion

The current study identified the differences of EBCDP knowledge, access, decision-making, and repository and quality improvement process use between the two middle- and two high-income countries. The distinct differences in EBCDP among the countries in this study can contribute to informing and improving global strategies for EBCDP implementation. Such efforts could take the form of cross-country conferences, as well as formal and informal partnerships where resources and knowledge could be shared. Political, administrative, and stakeholder leaders in these countries can also use these findings to glean valuable insight into areas that their public health practitioners need more assistance. Finally, findings from this study suggest that middle-income countries would benefit from increased funding, capacity building, and EBCDP infrastructure, to improve overall practitioner engagement in the implementation of EBCDP interventions.

## Publicly available datasets

The dataset used for this study is available in the publicly available Harvard Dataverse Repository at the following link: https://dataverse.harvard.edu/dataset.xhtml?persistentId=doi: 10.7910/DVN/HPJDGD.

## Ethics statement

All procedures performed in studies involving human participants were in accordance with the ethical standards of the institutional and/or National Research Committee and with the 1964 Helsinki declaration and its later amendments or comparable ethical standards.

## Author contributions

AD and XY contributed to the conception and design of the study, analysis and interpretation of the data, and drafting and revisions of the full manuscript. EB and KF contributed to the conception and design of the study, interpretation of data, drafting and revisions of the full manuscript. RR, ZW, PS-C, RA, TP, LB, TM, JS, TS, and RB contributed to the conception and design of the study and manuscript revisions and approval. All authors read and approved the final manuscript.

### Conflict of interest statement

The authors declare that the research was conducted in the absence of any commercial or financial relationships that could be construed as a potential conflict of interest.
