# Supplementary material for: Comparing Knowledge, Accessibility, and Use of Evidence-Based Chronic Disease Prevention Processes Across Four Countries
Source: Front Public Health. 2018 Aug 2;6:214. doi: 10.3389/fpubh.2018.00214 (PMC6095058; doi:10.3389/fpubh.2018.00214)
Supplement: Supplementary file 1 [file Data_Sheet_1.docx]

**Appendix A**

Table A1. Knowledge, use, and access related to evidence-based interventions in Australia, Brazil, China, and the United States in 2015-2016 (long version)

| **Variable** | **Australia (n=121)** | **Brazil (n=76)** | **China (n=102)** | **United States (n=101)** | **P value** |
| --- | --- | --- | --- | --- | --- |
| Evidence-based public health is defined as: “the process of integrating science-based interventions with community preferences to improve the health of populations” (Kohatsu et. al, 2004).With this definition in mind, how knowledgeable are you with evidence-based processes? (M ± SD) | 3.84±0.8 | 3.71±0.9 | 2.59±1.0 | 4.05±0.8 | <.001 |
| When you make decisions about such things as program planning and implementation, policy development, or funding, which of the following are important to you? (Select top three) |  |  |  |  |  |
| Support from leadership at my agency | 41(34%) | 59(78%) | 64(63%) | 35(35%) | <.001 |
| Support from elected officials | 7(6%) | 27(36%) | 42(41%) | 7(7%) | <.001 |
| Support from community partnerships | 55(46%) | 51(67%) | 42(41%) | 41(41%) | .001 |
| Recommendations from the funding agency | 3(3%) | 43(57%) | 18(17%) | 23(23%) | <.000 |
| Colleagues are using the intervention | 4(3%) | 53(70%) | 11(10%) | 3(3%) | <.001 |
| Available resources (program dollars & staff) | 51(42%) | 69(91%) | 53(52%) | 63(62%) | <.001 |
| How easy the intervention or policy is to implement | 6(5%) | 27(36%) | 51(50%) | 3(3%) | <.001 |
| Evidence regarding the effectiveness of the intervention | 89(74%) | 59(78%) | 23(23%) | 62(61%) | <.001 |
| Health planning tools | 13(11%) | 52(68%) | 13(13%) | 3(3%) | <.001 |
| Relevance of the intervention to the population of interest | 68(56%) | 65(86%) | 33(32%) | 47(47%) | <.001 |
| Seriousness of the health problem | 17(14%) | 59(78%) | 18(18%) | 11(11%) | <.001 |
| I have used repositories to find evidence-based interventions: (select one) |  |  |  |  | <.001 |
| in none of my programmatic areas | 8(7%) | 2(3%) | 30(29%) | 5(5%) |  |
| in a few of my programmatic areas | 29(24%) | 15(20%) | 51(50%) | 34(34%) |  |
| in many of my programmatic areas | 51(42%) | 34(45%) | 14(14%) | 47(47%) |  |
| in all of my programmatic areas | 23(19%) | 24(32%) | 4(4%) | 11(11%) |  |
| Staff at my agency use repositories of evidence-based interventions: (select one) |  |  |  |  | <.001 |
| in none of their programmatic areas | 1(1%) | 3(4%) | 24(24%) | 3(3%) |  |
| in a few of their programmatic areas | 28(24%) | 22(29%) | 43(43%) | 35(35%) |  |
| in many of their programmatic areas | 49(4%) | 33(43%) | 18(18%) | 42(42%) |  |
| in all of their programmatic areas | 12(10%) | 14(18%) | 1(1%) | 4(4%) |  |
| Staff at my agency use quality improvement processes: (select one) |  |  |  |  | <.001 |
| in none of their programmatic areas | 1(1%) | 4(5%) | 20(20%) | 1(1%) | <.001 |
| in a few of their programmatic areas | 25(21%) | 25(32%) | 46(46%) | 39(39%) |  |
| in many of their programmatic areas | 59(49%) | 34(44%) | 25(25%) | 50(50%) |  |
| in all of their programmatic areas | 25(21%) | 10(13%) | 2(2%) | 7(7%) |  |
| What avenues do you use to learn about the current study findings on evidence-based chronic disease prevention interventions? (select all that apply) |  |  |  |  |  |
| Academic journals | 107(88%) | 41(54%) | 57(56%) | 45(45%) | <.001 |
| CD-ROMs | 5(4%) | 3(4%) | 3(3%) | 3(3%) | .95 |
| Conferences | 92(76%) | 28(37%) | 29(28%) | 70(70%) | <.001 |
| Email alerts | 78(65%) | 15(20%) | 12(12%) | 59(59%) | <.001 |
| Evidence-based repositories | 57(47%) | 41(54%) | 16(16%) | 60(60%) | <.001 |
| Facebook | 13(11%) | 6(8%) | 24(24%) | 7(7%) | .001 |
| Funders | 15(10%) | 3(4%) | - | 42(42%) | - |
| Government agency staff | 32(26%) | 31(41%) | 0(0%) | 43(43%) | <.001 |
| Government reports | 76(63%) | 42(55%) | 5(5%) | 44(44%) | <.001 |
| Internet search engines | 74(61%) | 43(57%) | 30(29%) | 59(59%) | <.001 |
| Listservs/Newsletters/Online forums | 46(38%) | 4(5%) | 9(9%) | 49(49%) | <.001 |
| Media campaigns/Media interviews | 14(12%) | 10(13%) | 10(10%) | 11(11%) | .92 |
| Networks | 71(59%) | 18(24%) | 11(10%) | 28(28%) | <.001 |
| Partnerships | 81(67%) | 32(42%) | 18(18%) | 66(66%) | <.001 |
| Policy briefs | 36(30%) | 26(34%) | - | 32(32%) | - |
| Press releases | 23(19%) | 7(9%) | 11(11%) | 20(20%) | .08 |
| Twitter | 13(11%) | 0(0%) | - | 3(3%) | - |
| Stakeholders | 34(28%) | 14(18%) | - | 27(27%) | - |
| Technical assistance/Data liaison | 7(6%) | 22(29%) | 3(3%) | 11(11%) | <.001 |
| Trainings/Workshops/Meetings within my agency | 55(46%) | 27(36%) | 36(36%) | 42(42%) | .36 |
| Webinars | 56(46%) | 2(3%) | 2(2%) | 64(64%) | <.001 |
| For which avenues would you like additional access? (select all that apply) |  |  |  |  |  |
| Academic journals | 42(40%) | 39(51%) | 37(36%) | 29(32%) | .06 |
| CD-ROMs | 2(2%) | 5(7%) | 4(4%) | 2(2%) | .32 |
| Conferences | 26(25%) | 42(55%) | 21(21%) | 28(31%) | <.001 |
| Email alerts | 15(14%) | 8(11%) | 22(22%) | 13(14%) | .21 |
| Evidence-based repositories | 40(38%) | 17(22%) | 38(37%) | 32(35%) | .12 |
| Facebook | 4(4%) | 2(3%) | 25(25%) | 6(7%) | <.001 |
| Funders | 9(9%) | 7(9%) | - | 15(17%) | - |
| Government agency staff | 13(12%) | 11(15%) | 5(5%) | 7(8%) | .12 |
| Government reports | 19(18%) | 13(17%) | 9(9%) | 6(7%) | .04 |
| Internet search engines | 6(6%) | 9(12%) | 22(22%) | 6(7%) | .001 |
| Listservs/Newsletters/Online forums | 10(9%) | 9(12%) | 18(18%) | 15(17%) | .29 |
| Media campaigns/Media interviews | 6(6%) | 4(5%) | 7(7%) | 8(9%) | .78 |
| Networks | 20(19%) | 16(21%) | 3(3%) | 13(14%) | .001 |
| Partnerships | 34(32%) | 23(30%) | 21(21%) | 20(22%) | .17 |
| Policy briefs | 12(11%) | 5(7%) | - | 13(14%) | - |
| Press releases | 7(7%) | 5(7%) | 9(9%) | 6(7%) | .91 |
| Twitter | 4(4%) | 1(1%) | - | 3(3%) | - |
| Stakeholders | 9(9%) | 4(5%) | - | 4(4%) | - |
| Technical assistance/Data liaison | 24(23%) | 6(8%) | 14(14%) | 18(20%) | .04 |
| Trainings/Workshops/Meetings within my agency | 27(26%) | 24(32%) | 36(35%) | 20(22%) | .17 |
| Webinars | 25(21%) | 3(4%) | 6(6%) | 19(21%) | <.001 |

^a^ Cells with - indicate that response option was not available in a given country’s survey due to contextual irrelevance.
